# Contactless facial video recording with deep learning models for the detection of atrial fibrillation

**DOI:** 10.1038/s41598-021-03453-y

**Published:** 2022-01-07

**Authors:** Yu Sun, Yin-Yin Yang, Bing-Jhang Wu, Po-Wei Huang, Shao-En Cheng, Bing-Fei Wu, Chun-Chang Chen

**Affiliations:** 1 Department of Neurology, En Chu Kong Hospital, New Taipei City, Taiwan, ROC; 2 Institute of Electrical and Control Engineering, National Yang Ming Chiao Tung University, 1001 University Road, Hsinchu, 30010 Taiwan, ROC; 3 Department of Cardiology, New Taipei City Hospital, New Taipei City, Taiwan, ROC

**Keywords:** Computational biology and bioinformatics, Cardiology, Neurology

## Abstract

Atrial fibrillation (AF) is often asymptomatic and paroxysmal. Screening and monitoring are needed especially for people at high risk. This study sought to use camera-based remote photoplethysmography (rPPG) with a deep convolutional neural network (DCNN) learning model for AF detection. All participants were classified into groups of AF, normal sinus rhythm (NSR) and other abnormality based on 12-lead ECG. They then underwent facial video recording for 10 min with rPPG signals extracted and segmented into 30-s clips as inputs of the training of DCNN models. Using voting algorithm, the participant would be predicted as AF if > 50% of their rPPG segments were determined as AF rhythm by the model. Of the 453 participants (mean age, 69.3 ± 13.0 years, women, 46%), a total of 7320 segments (1969 AF, 1604 NSR & 3747others) were analyzed by DCNN models. The accuracy rate of rPPG with deep learning model for discriminating AF from NSR and other abnormalities was 90.0% and 97.1% in 30-s and 10-min recording, respectively. This contactless, camera-based rPPG technique with a deep-learning model achieved significantly high accuracy to discriminate AF from non-AF and may enable a feasible way for a large-scale screening or monitoring in the future.

## Introduction

As the world’s population is ageing, atrial fibrillation (AF) has become a serious public health issue. Patients with AF-related ischemic stroke were more likely to have severe disability, high recurrence rate, high fatality rate, and greater medical cost than those without^1–3^. Because of its paroxysmal and asymptomatic natures^4,5^, AF is commonly diagnosed after an ischemic stroke has occurred^6^. Anticoagulants can significantly reduce the risk but documentation of AF is required to initiate this preventive therapy. Thus screening for AF in particularly among the elderly is recommended^7,8^.

Current methods of AF detection like implanted loop recorder^9,10^ and electrocardiogram (ECG) patch^11^ are either invasive or expensive, while handheld recorder^12^ is convenient for screening but not feasible for long-term monitoring. Photoplethysmography (PPG) with pulse waveforms generated from optical sensors of mobile devices has become a new trend and shows sufficient accuracy for the detection of heart rate and other physiological parameters^13^. Recent studies revealed various algorithms with good performance in discriminating AF from sinus rhythm^14–17^. Though digital wearables are increasingly popular worldwide, most elderly, who are the main population of AF, are still not used to the application of this high-tech device^18^. An emerging noncontact technique called remote photoplethysmography (rPPG) has been developed for detecting heart rate, which uses digital camera to measure the subtle variations of skin color reflecting the cardiac pulsatile signal due to heart activity pumping blood to and from the face^19–21^. This method would potentially be applied to mass screen with less cost, as well as to long-term monitoring under appropriate settings. Studies in estimating the accuracy of rPPG in detecting AF are still limited^22,23^. In this study, we sought to estimate the ability of rPPG measurement with deep learning (DL) models in discriminating AF from non-AF.

## Methods

### Study population and examination procedure

This was a prospective, single-center study conducted between June 1, 2019 and August 31, 2020 with participants recruited from outpatient departments of neurology and cardiology, and neurological ward at En Chu Kong Hospital, New Taipei City, Taiwan. Patients in critical medical conditions were excluded. All participants provided written informed consent prior to their enrollment in the study. This study followed the tenets of the Declaration of Helsinki and was approved by the Institutional Review Board of En Chu Kong Hospital (ECKIRB10803006). All the methods were performed in accordance with the relevant guidelines and regulations. In the examination room, each participant received a standard 10-s, 12-lead ECG and then immediately sat in front of a digital camera at a distance of 1–2 m for facial video recording. We placed another 3-lead ECG monitor (Deluxe-100, North-vision Tech. Inc. Taiwan) which was simultaneously started with the video recording. Participants were instructed to position themselves as stable as possible and to minimize movement during recording. The ambient light source for recording is the daylight lamp where the illuminance measured in front of participants’ face was at around 200–400 lx. The video recording ended at 10 min or when subjects declined to continue before that time point.

### ECG diagnosis and facial rPPG recording

Participants were classified into three groups based on their 12-lead ECG results: first, “AF” with or without other abnormal ECG patterns; second, “normal sinus rhythm” (NSR) in which the ECG results were completely normal; third, “Others” with abnormal ECG results except AF. In this study, we assumed that the presence of AF in patients would persist during the periods of receiving the 12-lead ECG exam and subsequent 10 min of facial video-recording. The 12-lead ECG data were analyzed by a cardiologist blinded to the rPPG results.

**Figure 1:**
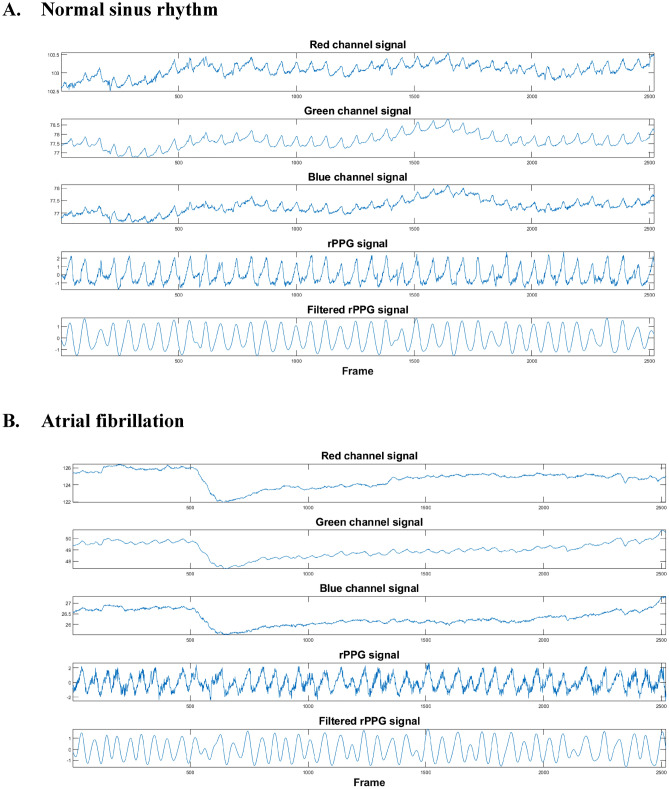

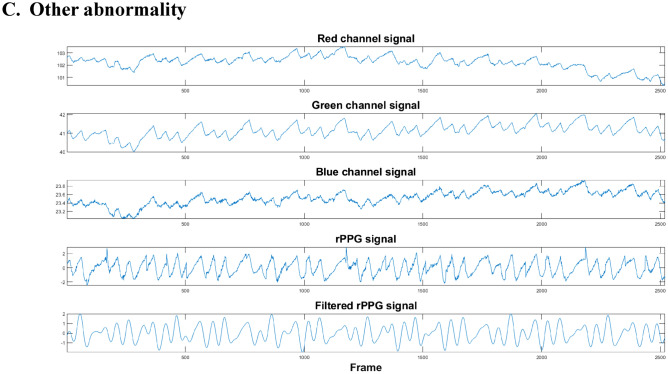
The examples of extracted RGB and rPPG signals. (**A**) Signals of a subject with normal sinus rhythm. (**B**) Signals of a subject with atrial fibrillation. (**C**) Signals of a subject with atrial premature complexes.

In order to obtain the heart rhythm information from the facial image sequence, we used industrial camera (84 frames per second in VGA resolution) (FLIR BFLY-U3-03S2C-CS) as a sensor to capture face images. Once the image was captured, the face detection algorithm^24^ was used to locate the region of interest (ROI) on the face. Then, we averaged the RGB value from the ROI and used an optical technique^25^, rPPG- to capture the subtle color change of the skin due to the blood pulsation caused by the heartbeat. To reduce the noise caused by motion or environment illuminance variance, a forth order Chebyshev II bandpass filter (cutoff frequency: 0.5–3 Hz) was utilized. The example of extracted RGB signal, original rPPG, and filtered rPPG was shown in Fig. 1. It should be noticed that the camera setting about the auto exposure, auto gain, and auto focus were all disabled to ensure the rPPG signal quality. The entire recording of rPPG signals of each participant was divided into multiple 30-s segments. If the subject was classified as AF, his or her total segments of rPPG data would be labeled as AF, and the same rule applied to groups of “NSR” and “Others”.

### Model development and AF prediction

**Figure 2:**
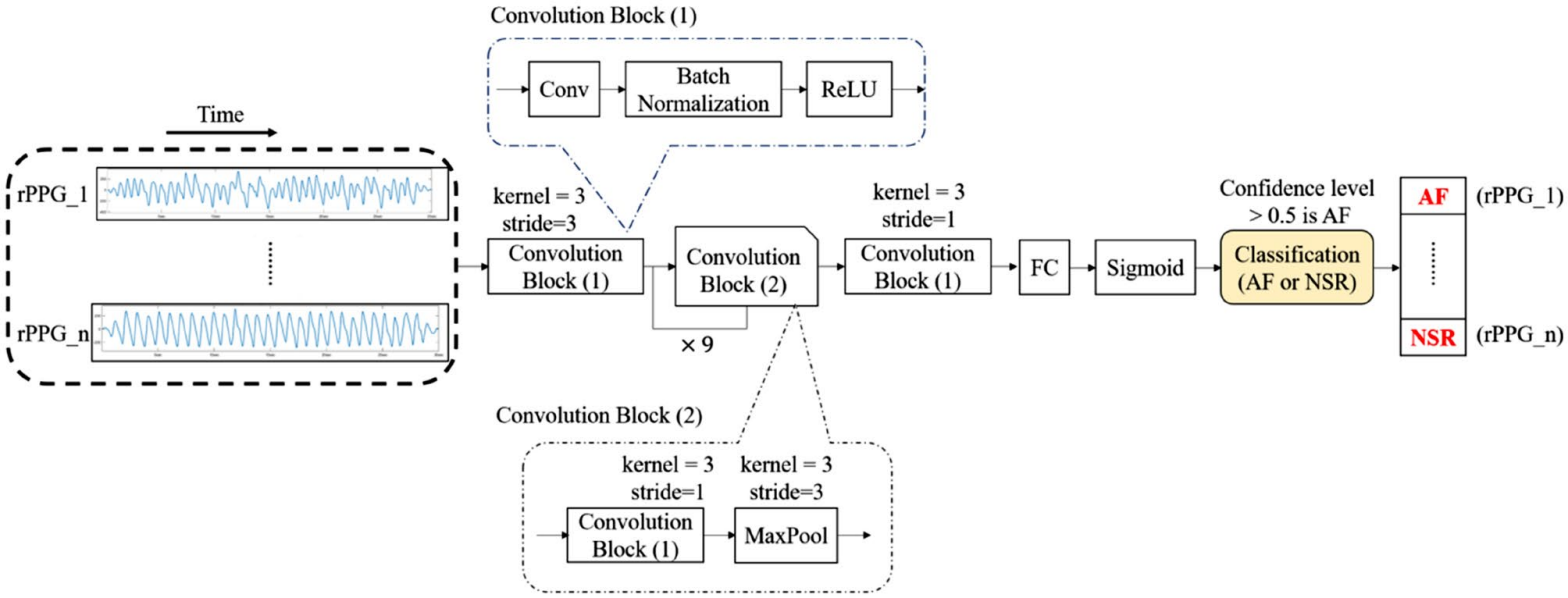
The overall DCNN model architecture.

We adopted a sample-level, 12-layer, deep convolutional neural networks (DCNN)^26^ fed with 30-s segments of rPPG signals as the input for the feature computation. The overall model architecture was shown in Fig. 2 and Supplementary Fig. S1. For the first 11 convolutional layers, batch normalization, dropout and max pooling were included in several different layers. As for the activation function between convolutional layers, a rectified linear activation function was applied. A fully-connected layer was used as the last layer. The model was designed as a binary classifier, using annotations of ECG from a cardiologist as ground truth and the probabilities of predictions as output.

**Figure 3:**
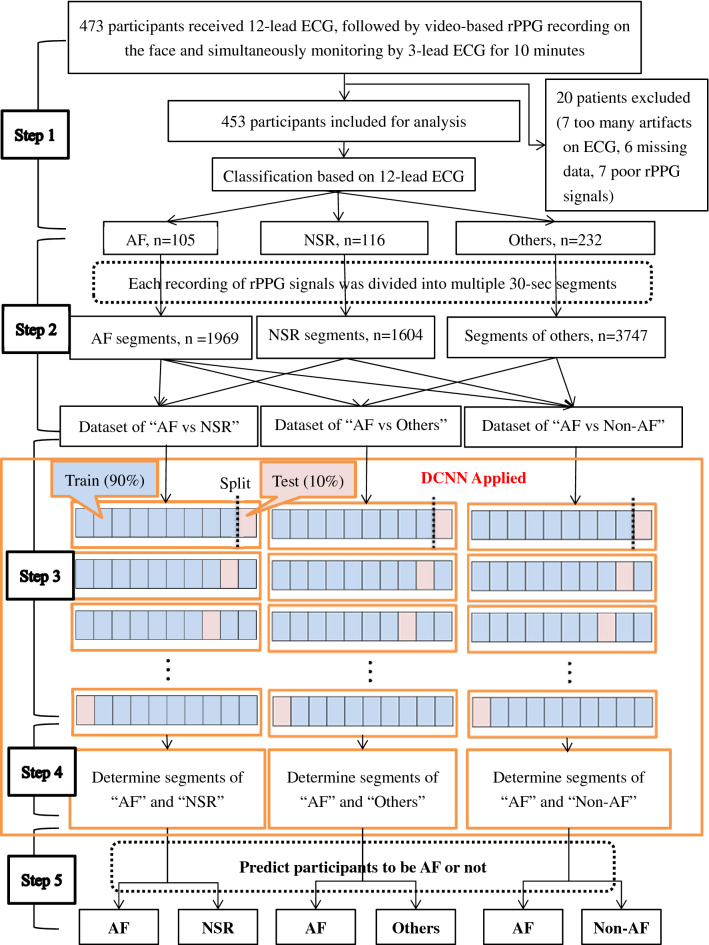
Study flow diagram. *AF* atrial fibrillation, *DCNN* deep convolutional neural network, *ECG* electrocardiograph, *NSR* normal sinus rhythm, *rPPG* remote photoplethysmography. Step 1: Case enrollment and ECG-proved classification. Step 2: Extraction of rPPG signals and dividing them into 30-s segments as the data of for three datasets: “AF vs NSR”, “AF vs Others”, “AF vs Non-AF”. Step 3: Each segment was used as the input of DCNN learning model. For each dataset, tenfold cross validation method was applied to measure the performance of the models with data split into train set (9 folds) and test set (onefold). The procedure was repeated 10 times until all folds had served exactly once as the hold-out set. Eventually, we calculated the average accuracy of the ten folds as the performance of the model and the standard deviation values of model performance between each fold were also calculated. Step 4: Best model algorithms were generated to determine whether or not the 30-s-rPPG segment to be AF. Step 5: Participant with more than 50% of segments determined as AF by the above models was considered positive for AF.

The DCNN was implemented with PyTorch framework for model training in three datasets: “AF vs NSR”, “AF vs Others” and “AF vs Non-AF”, separately. In order to apply the DL algorithms to the population with various heart conditions, three datasets were formed and DCNN models were trained with input data of 30-s segments to discriminate AF from NSR, from other abnormalities, and from all non-AF, respectively. The data of rPPG segments from each dataset was partitioned into 10 equal-sized sets and we used tenfold cross-validation method for model training and validation^27^. The next step was to detect AF patients. Participants with 10 min of video recording would be supposed to have 20 segments of 30-s length. Every segment is fed into the model to obtain an atrial fibrillation score by voting algorithm^28^. If the score is bigger than 0.5, the corresponding participant would be classified as an AF case. Figure 3 showed the whole process of the study.

### Statistical analysis

The diagnostic performance of the DCNN model was evaluated by calculating the sensitivity, specificity, positive predictive value, and accuracy rate using the confusion matrix with 12-lead ECG as the reference standard. We plotted receiver operating characteristic (ROC) curve and measured the area under the curves (AUC) to verify the performance of the binary classifier system under discrimination threshold. Since the models were trained in three datasets: “AF vs NSR”, “AF vs Others”, and “AF vs all Non-AF”, calculation of the measures was performed for these three models respectively. For estimating the diagnostic accuracy of whole length of rPPG recording on each subject, we repeated the calculations of AUC/sensitivity/specificity/ positive predictive value/accuracy rate for these three datasets with AF participants identified by voting algorithm. A sensitivity analyses was performed to check the relationship between camera recording time and the accuracy rate of rPPG in detecting AF based on 15- to 300-s data segments.

## Results

Of the 473 enrolled patients, 20 were excluded due to the following reasons: 7 had too many artifacts on ECG, 6 had missing data on ECG or on facial recordings, and 7 had the quality of rPPG signals too poor to read. Finally, we have a total of 453 (mean age, 69.3 ± 13.0 years, women, 46%) patients successfully analyzed, in which the mean length (± standard deviation, SD) of video recording for each participant was 484 (± 148) seconds and the average number (± SD) of 30-s samples per person was 16.12 (± 4.93). Based on 12-lead ECG, there were 116 participants with NSR, 105 with AF, and 232 classified as others. The patients with AF (mean age, 74.3 ± 12.5 years, women, 51.4%) were older with more women than patients without AF (mean age, 67.8 ± 13.0 years, women, 44.8%). The ECG patterns in the group “Others” included abnormalities in rate and rhythm (e.g., sinus arrhythmia, atrial premature complexes, atrial flutter, ventricular premature complex), axis (e.g., right or left axis deviation), amplitude (e.g., ST depression, T-wave abnormality), durations and intervals (e.g., conduction delay or block, right or left bundle branch block, long QT). Pacing was found on the ECG of 5 participants of whom 2. Besides, the ECG patterns of 16 patients, though manually interpreted as snus rhythm, were classified into the group of “Others” because their ECG resembled arrhythmia or atypical morphology of NSR due to various artifacts. The various ECG patterns of the participants of group “Others” were summarized in Table S1 in the Supplementary Information. Individuals with both AF and other ECG abnormalities were classified as “AF” group and were not uncommon in the study population.

**Figure 4:**
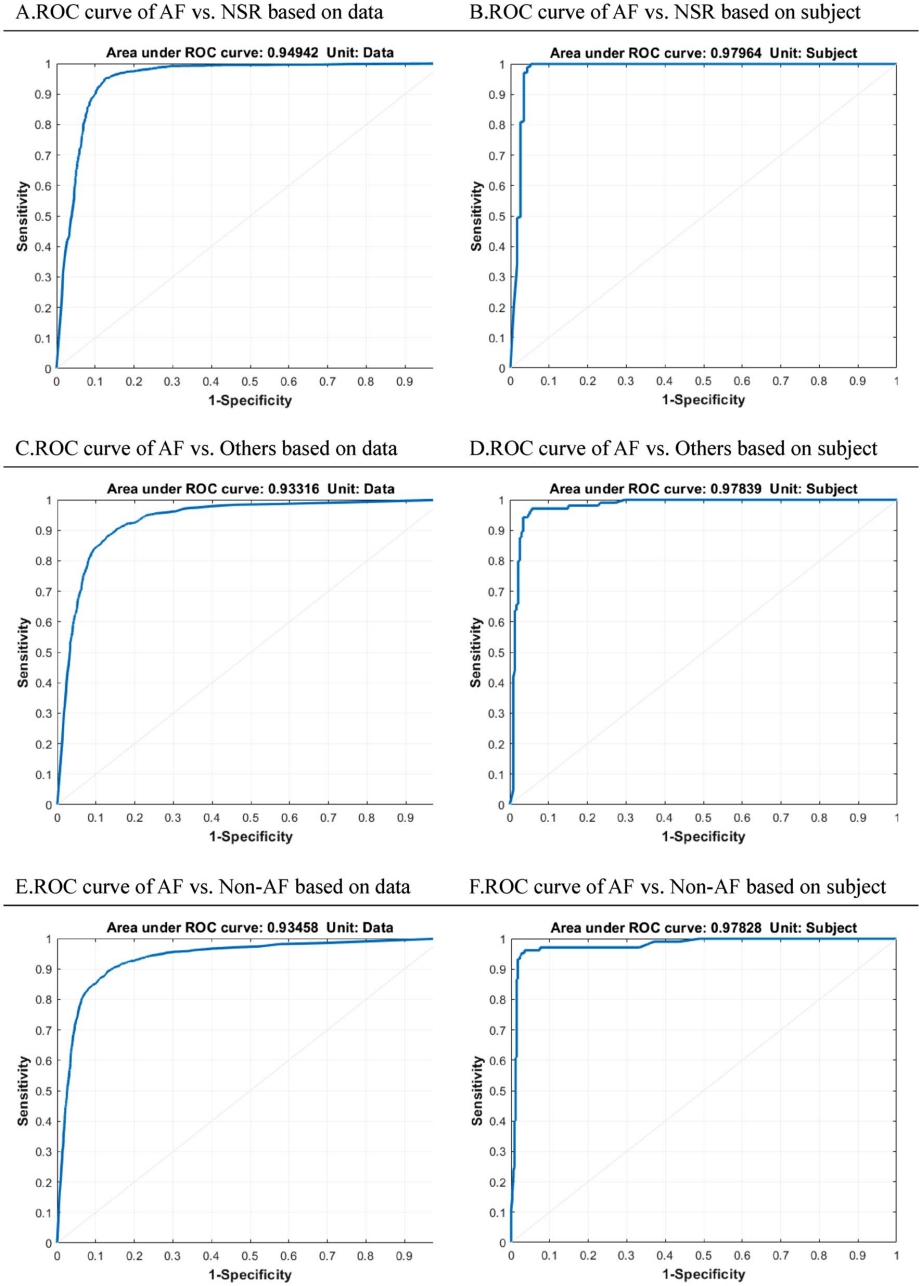
Performance of the deep-learning models for classification of atrial fibrillation based on 30-s segment data and whole-length recording of subjects. *AF* atrial fibrillation, *NSR* normal sinus rhythm, *ROC* receiver operating characteristic. Receiver operating characteristic (ROC) curves based on data of 30-s segments (Left) and voting results of whole-length recording of subjects (Right) in the datasets: “AF vs NSR” (Fig. 2A,B), “AF vs Others” (Fig. 2C,D), “AF vs Non-AF” (Fig. 2E,F). By voting algorithm, subject who had more than 50% of model-determined AF segments was classified as AF case.

This DCNN model had gained great success in the audio processing region since the relatively slim architecture and smaller convolution kernel were applied, which were also suit for the rPPG signal processing. We separately plotted the ROC curves of “AF vs. NSR”, “AF vs. Others” and “AF vs. Non-AF” of the three models based on data of 30-s rPPG segments (Fig. 4A,C,E). The ROC curves were plotted again based on the aforementioned voting results for predicting subject to be AF or not (Fig. 4B,D,F). The results of the performance of models with measures of AUC, sensitivity, specificity, positive predictive value, and accuracy rates were shown in Table 1. The performance of DL algorithms changed as the model was trained in different population composition. For predicting AF by 30-s-rPPG segments in the dataset of “AF vs NSR”, the sensitivity, specificity, positive predictive value and accuracy rates of test set were 95.0%, 87.3%, 90.2% and 91.6%, respectively. The diagnostic sensitivity and accuracy decreased while the specificity slightly increased as the model was trained in the dataset of “AF vs Others”. With all data pooled together, the sensitivity, specificity, positive predictive value and accuracy rates in discriminating AF from non-AF by the algorithm were 80.3%, 93.6%, 82.1% and 90.0%, respectively (Table 1). We checked the relationship between video recording time and the accuracy rate of rPPG and found that the accuracy rate reached a peak when the segment length was set at 120–240 s. This results were shown in Supplementary Fig. S2. We further calculated the diagnostic accuracy by assessing the whole length of rPPG recording signals for each subject. For the model trained in the dataset of “AF vs NSR”, the sensitivity, specificity, positive predictive value and accuracy rates on the test set were 99.1%, 94.8%, 94.6% and 96.8%, respectively. For the model trained in the dataset of “AF vs Others”, though the sensitivity slightly decreased, the algorithm still performed well with the sensitivity, specificity, positive predictive value and accuracy being 94.3%, 95.7%, 90.8% and 95.3%, respectively. Finally, we evaluated the model performance in detecting AF among all participants with and without arrhythmia or ECG abnormalities. The results showed high sensitivity (93.3%) and high positive predictive value (94.3%). The accuracy and specificity rates were even up to 97.1% and 98.3%, respectively (Table 1). The corresponding values for the performance of training datasets and testing datasets were shown in Table S2 and Table S3 in the Supplementary Information.

**Table 1:** Confusion matrix for classification of atrial fibrillation based on data of 30-s segments (n = 7320) and on voting results of whole recording on each subject (n = 453).

Validation data sets	Value %				
	AUC	Sensitivity	Specificity	PPV	Accuracy
**Data of segments**					
AF vs NSR	0.93 ± 0.2	95.0 ± 3.3	87.3 ± 6.4	90.2 ± 3.9	91.6 ± 2.4
AF vs Others	0.95 ± 0.3	83.8 ± 7.7	90.6 ± 3.8	82.4 ± 6.3	88.2 ± 2.8
AF vs Non-AF	0.93 ± 0.0	80.3 ± 10.0	93.6 ± 1.9	82.1 ± 5.3	90.0 ± 3.0
**Subject**					
AF vs NSR	0.98 ± 0.0	99.1 ± 0.8	94.8 ± 3.0	94.6 ± 1.6	96.8 ± 1.5
AF vs Others	0.98 ± 0.0	94.3 ± 3.2	95.7 ± 2.6	90.8 ± 4.1	95.3 ± 2.0
AF vs Non-AF	0.98 ± 0.0	93.3 ± 5.9	98.3 ± 1.5	94.3 ± 3.7	97.1 ± 2.4

*AF* atrial fibrillation, *AUC* area under the curve, *ECG* electrocardiograph, *NSR* normal sinus rhythm, *PPV* positive predictive value, *rPPG* remote photoplethysmography.

## Discussion

We demonstrated that a camera-based recording can detect AF using the rPPG technology incorporated with DL algorithms. With 12-lead ECG as standard reference, algorithm performance from 10 min of rPPG recording achieved high sensitivity (93.3%) and specificity rates (98.3%) with accuracy rate up to 97.1% in discriminating AF patients from those without AF. The ultrashort 30-s recording segment also yielded a high accuracy rate (90%). Even in the sample population of group “Others” with various abnormalities or arrhythmic ECG, the positive predictive value remained high (90.8%), which indicated low false positive rate. These data support the ability of DL-assisted rPPG to correctly discriminate AF from other pulse irregularity and abnormal ECG waveforms just by using a camera and even by a very short time recording.

In recent years, there have been several studies reporting the use of smartphones and their apps in detecting AF. These apps demonstrated good performance in detecting AF^15,16,29,30^, with accuracy rate around 95%-98% according to a review by Pereira et al.^15^, in which the smartphone camera recorded PPG signals through fingertip contact. Like handheld ECG recorder, the heart rhythm can only be measured as long as the person does not move the device because even slight movement may severely distort measurement. In addition, digital wearables may not be so popular among the less-tech savvy individuals, as well as the elderly who are really the high AF-risked population^18^. Other than the detection performed by direct contact on the devices, the rPPG method with video recording on the face has the pulsatile signals remotely captured. The motion-robust rPPG algorithms enable the recording with an affordable, consumer-level camera under “normal” ambient light conditions^19,31^, and even for multiple people at the same time with minimal motion distortions^32–34^. These advantages make rPPG a promising tool not just for mass screening of AF but also for the remote, long-term heart rate monitoring outside the hospital, such as at home, in workplace environment or even in driving condition^35^.

In terms of using video-based rPPG for AF detection, relatively low error rates (17–29%) in the study by Couderc and colleagues proved that this method is feasible^22^. Another two studies by Shi et al. and Eerikainen et al. with small sample size also showed promising results with accuracy rates improved to 92–98%^27,36^. A study by Yan and colleagues used smartphone camera for contactless facial recording and analyzed the data by the Cardio Rhythm application in patients with AF and sinus rhythm yielded 95% of sensitivity and specificity^16,23^. Our study extended the application of using facial rPPG to differentiate AF from not only normal sinus rhythm but also other arrhythmia and various ECG patterns and also proved the high accuracy rate (97%). Since the diversity and size of data are very important factors for better performance of DL algorithm, more than half (232/453) of our participants, as classified as group “Others”, were neither normal sinus rhythm nor AF. Their ECG patterns showed various abnormalities in rate, rhythm, axis, wave morphology, durations and intervals. The results of the algorithm performance differed between datasets fed with more “normal ECG” subjects and dataset with more “abnormal ECG” subjects. Algorithm for population with AF and NSR achieved highest sensitivity. The sensitivity decreased but the specificity increased when model trained by lots of abnormal ECG patterns as feature inputs. Among the 453 participants, there were a total of 36 subjects misclassified by the models as to be either false positive or false negative cases (7 in the model of “AF vs NSR”, 16 in “AF vs Others”, 13 in “AF vs Non-AF”). Interestingly, all of them were misclassified by only 1 of the 3 models. For example, subjects who were misclassified in the dataset composed of “AF and NSR” were not misclassified in the dataset composed of “AF and non-AF”, and vice versa, suggesting the results of DL markedly affected by population composition of ECG characteristics. Since the participants were enrolled from the stroke ward and the department of cardiology and neurology, most of the participants were old and many had heart disease or rhythm problems. Previous reports showed that some abnormal rhythms such as ventricular premature contractions, atrial premature contractions and sinus arrhythmia were likely to be false positive of AF by PPG-based wearables^16,18^. This study performed model training from various abnormal ECG patterns including the morphology of pacing. There were 2 AF patients among the 5 participants with pacing. These 2 AF patients were correctly classified by rPPG with deep-learning algorithm. But 1 of the other 3 patients without AF was misclassified as AF by the model. Overall, this study showed relatively low rate of misclassification by models among subjects with abnormal rhythms, e.g. 0/11 sinus tachycardia, 0/2 sinus bradycardia, 1/15 ventricular premature contractions, 0/11 atrial premature contractions, 3/15 sinus arrhythmia (2 in “AF vs Non-AF” model, 1 in “AF vs Others” model were misclassified among 15 sinus arrhythmia). And the ECG findings in our patients with AF not just showed AF alone. Instead, most of them have combined with other abnormal morphology or rhythms. DL approaches can yield good performance in detecting AF even in the population with high burden of other arrhythmia^37^. Algorithms developed by present three training models all achieved high accuracy rates (> 95%) in detecting AF patients, either from the datasets with more NSR or from that with more other arrhythmias or abnormal ECG features.

The Heart Rhythm Society consensus statement defined AF as an arrhythmia lasting ≥ 30 s on 1-lead ECG or if present on the entire 10 s 12-lead standard ECG^38,39^. This study estimated the performance of rPPG-based algorithms in both 30-s samples and 10 min of recordings. Though the current rule of 30-s recording cannot be applied on the model-predicted pulse irregularity presented on the form other than ECG, this rPPG-DL modality as with high sensitivity and low false positive rate on detecting AF shown in this study enables a promising tool for screening. Furthermore, the DL models achieved higher accuracy along with higher detection rate as the recording time increasing, which suggests a favorable cost-effective option in calculating AF burden by using rPPG for long-term monitoring in the future.

The performance of DL algorithms changes when models are trained in datasets from different target subjects. Thus, algorithms developed in wearables such as smartphones among mostly young people may not be suitable for the elderly. Similarly, the best model trained in hospital setting may not be as good when applying in community screen. This study provides the evidence that rPPG-DL method may enable a reliable, non-contact screening or monitoring for AF detection as with the model specifically trained in the target individuals. The cut point of > 50% AF(+) 30-s samples in this study for predicting AF individual may also need to be adjusted to get better predictive ability when applying to another population.

### Limitations

There are some limitations to the study. First, the 12-lead ECG and video-based rPPG recording were not simultaneously performed. The depicted waveforms, in particular p-wave, of the output data from our simultaneous monitoring by 3-lead ECG were not clearly discernible in severe background noise. In addition, some tachycardia or ectopic beats on 3-lead monitor were also difficult in the interpretation of irregular rhythms. In order not to misclassify the participants with various cardiac diseases, we chose 12-lead ECG as standard reference. Since all of the subjects with AF in this study were either cases of long-term follow-up at our outpatient clinic, or cases of acute embolic stroke with high AF burden, it is highly possible that their AF would persist during the 10-s of 12-lead ECG exam and subsequent 10 min of rPPG recording. Nevertheless, the occurrence of paroxysmal AF during this 10-min examination still cannot be excluded. Second, all subjects in this study were selected to be able to position themselves sitting and facing camera steadily. There may be some selection bias as very ill patients were excluded. The applicability of rPPG on these kinds of patients is uncertain. Third, seven participants with poor rPPG recording were excluded due to too much motion artifacts and poor lighting. Lighting conditions and motion artifacts are always great challenges for rPPG recording. Though motion-robust rPPG algorithms are proposed in extracting a clean pulse signal in ambient light environments using a regular color camera in subjects who move significantly^32,33,40^, studies to examine the generalizability in monitoring subjects for AF detection under free-living condition are warranted. Fourth, because some of our elderly patients, especially those with cognitive impairment, were unable to tolerate sitting steadily for 10 min, the length of video-recording time among our participants varied from 5 to 10 min. Thus we could not precisely make the conclusion for how long the exact recording time should be to obtain the accuracy rate provided in this study. The last point is that we chose industrial camera for the purpose of obtaining high resolution image. Although motion-robust rPPG algorithms have been developed by using consumer-level camera in detecting heart rate^9,31^ and facial video recording by smartphone camera has been used to differentiate AF from NSR^16,23^, the ability of AF detection from other various abnormal ECG for mass screen by using regular camera needs further studies.

## Conclusions

The application of camera-based rPPG technique with DL algorithms achieved high accuracy in detecting AF among population with normal and various abnormal ECG patterns. The robust performance of deep learning models may enable rPPG, either by a common video camera or by a smartphone with built-in camera, to be a promising tool for mass screening and long-term monitoring in a non-contact and a favorable cost-effective way. This would represent a major advance for stroke prevention in the near future. Further studies are warranted to evaluate the model performance in hospital setting, large-scale community screening, and home-based long-term monitoring.

## Supplementary Information

Supplementary Information.
