# Supplementary material for: Contactless facial video recording with deep learning models for the detection of atrial fibrillation
Source: Sci Rep. 2022 Jan 7;12:281. doi: 10.1038/s41598-021-03453-y (PMC8741942; doi:10.1038/s41598-021-03453-y)

**Supplementary Information**

**Contactless facial video recording with deep learning models for the detection of atrial fibrillation**

Yu Sun^1^, Yin-Yin Yang^2^, Bing-Jhang Wu^2^, Po-Wei Huang^2^, Shao-En Cheng^2^, Bing-Fei Wu^2*^, Chun-Chang Chen^3^

^1^Department of Neurology, En Chu Kong Hospital, New Taipei City, Taiwan

^2^Institute of Electrical and Control Engineering, National Yang Ming Chiao Tung University, Hsinchu, Taiwan

^3^Department of Cardiology, New Taipei City Hospital, New Taipei City, Taiwan

***Corresponding Author:** Bing-Fei Wu

Institute of Electrical and Control Engineering, National Yang Ming Chiao Tung University, 1001 University Road, Hsinchu, Taiwan, 30010

Email: [bwu@cssp.cn.nctu.edu.tw](mailto:bwu@cssp.cn.nctu.edu.tw), TEL: 886-35131538

| **Table S1. ECG Abnormalities in 232 Participants Classified as "Others", with ECG Pattern other than Normal Sinus Rhythm and Atrial Fibrillation (AF)** | | | |
| --- | --- | --- | --- |
| Abnormal ECG patterns | | N | %^a^ |
| Rate and rhythm | |  |  |
|  | sinus tachycardia | 11 | 4.7 |
|  | sinus bradycardia | 3 | 1.3 |
|  | sinus arrhythmia | 15 | 6.5 |
|  | atrial premature complex | 11 | 4.7 |
|  | atrial rhythm | 5 | 2.2 |
|  | atrial flutter | 3 | 1.3 |
|  | Wolff-Parkinson-White syndrome | 1 | 0.4 |
|  | ventricular premature complex | 15 | 6.5 |
| Axis | |  |  |
|  | left axis deviation | 22 | 9.5 |
|  | right axis deviation | 3 | 1.3 |
| Amplitude, duration and interval | |  |  |
|  | atrioventricular block | 36 | 15.5 |
|  | intraventricular conduction delay | 9 | 3.9 |
|  | right bundle branch block | 33 | 14.2 |
|  | left bundle branch block | 2 | 0.9 |
|  | left anterior fascicular block | 4 | 1.7 |
|  | T-wave abnormality | 27 | 11.6 |
|  | ST depression | 36 | 15.5 |
|  | non-specific T-wave abnormality | 74 | 31.9 |
|  | long QT | 23 | 9.9 |
|  | low QRS | 1 | 0.4 |
|  | left ventricular hypertrophy | 21 | 9.1 |
|  | myocardial infarction | 21 | 9.1 |
| Others | |  |  |
|  | Pacemaker ^b^ | 3 | 1.3 |
|  | atypical pattern ^c^ | 16 | 6.9 |
| AF, atrial fibrillation; ECG, electrocardiograph | | | |
| ^a^ The denominator is 232, the number of participants classified as "Others".  ^b^ Pacing was found on the ECG of 5 participants, of whom 2 have AF.  ^c^ The ECG patterns resembled arrhythmia or abnormal waveform morphology due to some artifacts, but those were finally confirmed by a cardiologist as normal pattern. | | | |
|  | | | |
|  | | | |

| **Table S2. Performance of Deep Learning Models in Discriminating rPPG Segments of AF (n=1969) from those of Normal Sinus Rhythm (n=1604) and those of Other Abnormality (n=3747) in Training Datasets and Testing Datasets** |
| --- |
| \| Value \| AF vs NSR \| \| AF vs Others \| \| AF vs Non-AF \| \| \| --- \| --- \| --- \| --- \| --- \| --- \| --- \| \| Train \| Test \| Train \| Test \| Train \| Test \| \| Sensitivity, % \| 98.7 ± 3.2 \| 95.0 ± 3.3 \| 95.3 ± 9.3 \| 83.8 ± 7.7 \| 97.8 ± 3.1 \| 80.3 ± 10.0 \| \| Specificity, % \| 98.6 ± 3.0 \| 87.3 ± 6.4 \| 98.6 ± 2.4 \| 90.6 ± 3.8 \| 99.2 ± 1.7 \| 93.6 ± 1.9 \| \| PPV, % \| 98.8 ± 2.5 \| 90.2 ± 3.9 \| 97.3 ± 4.7 \| 82.4 ± 6.3 \| 97.8 ± 4.2 \| 82.1 ± 5.3 \| \| Accuracy, % \| 98.6 ± 3.1 \| 91.6 ± 2.4 \| 97.5 ± 4.0 \| 88.3 ± 2.8 \| 98.8 ± 1.6 \| 90.0 ± 3.0 \|   AF, atrial fibrillation; NSR, normal sinus rhythm; PPV, positive predictive value; rPPG, remote photoplethysmography |
|  |
|  |
|  |

| **Table S3. Performance of Deep Learning Models in Discriminating Participants with AF (n=105) from those with Normal Sinus Rhythm (n=116) and those with Other Abnormalities (n=232) in Training Datasets and Testing Datasets** |
| --- |
| \| Value \| AF vs NSR \| \| AF vs Others \| \| AF vs Non-AF \| \| \| --- \| --- \| --- \| --- \| --- \| --- \| --- \| \| Train \| Test \| Train \| Test \| Train \| Test \| \| Sensitivity, % \| 99.9 ± 1.3 \| 99.1 ± 0.8 \| 97.3 ± 5.6 \| 94.3 ± 3.2 \| 99.3 ± 1.3 \| 93.3 ± 5.9 \| \| Specificity, % \| 99.8 ± 1.9 \| 94.8 ± 3.0 \| 99.7 ± 1.1 \| 95.7 ± 2.6 \| 99.9 ± 0.6 \| 98.3 ± 1.5 \| \| PPV, % \| 99.8 ± 1.9 \| 94.6 ± 1.6 \| 99.4 ± 2.3 \| 90.8 ± 4.1 \| 99.6 ± 1.9 \| 94.3 ± 3.7 \| \| Accuracy, % \| 99.8 ± 1.6 \| 96.8 ± 1.5 \| 99.0 ± 2.2 \| 95.3 ± 2.0 \| 99.8 ± 0.7 \| 97.1 ± 2.4 \|   AF, atrial fibrillation; NSR, normal sinus rhythm; PPV, positive predictive value; rPPG, remote photoplethysmography |
|  |
|  |

**Figure S1. Layers of the Architecture of Deep Neural Network Model**

**
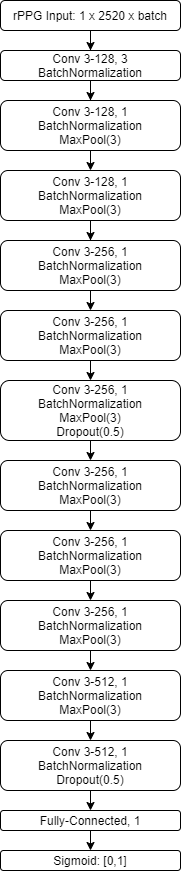
**

**Figure S2. The relationship between camera recording time and the accuracy rate of rPPG in detecting AF based on 15- to 300-second data segments.**


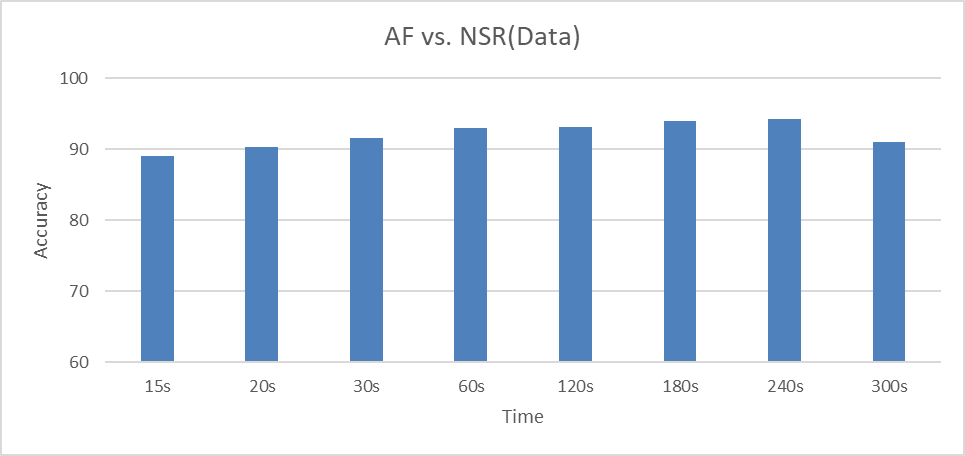


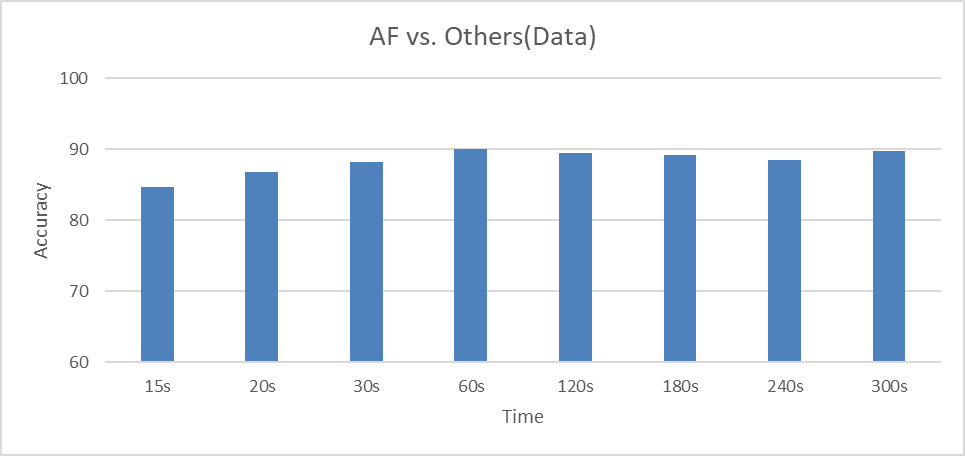


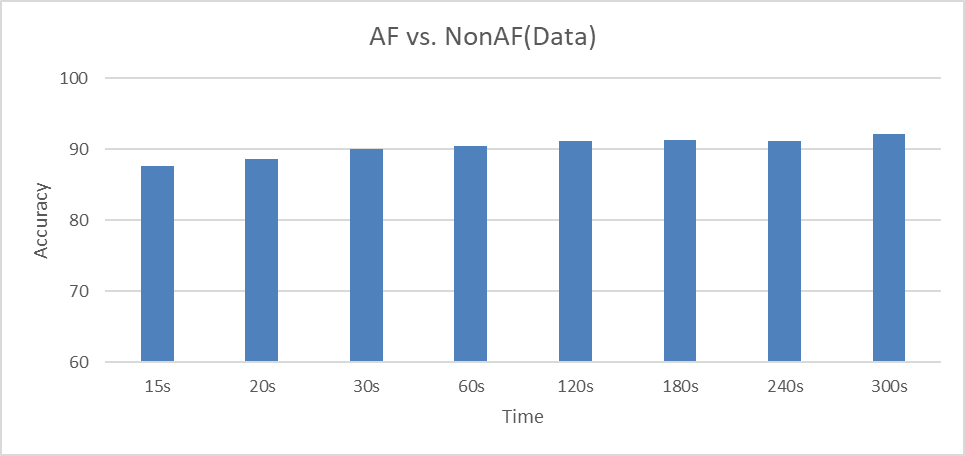

Supplement: Supplementary file 1 — Supplementary Information. [file 41598_2021_3453_MOESM1_ESM.docx]
